# Association of Protein Translation and Extracellular Matrix Gene Sets with Breast Cancer Metastasis: Findings Uncovered on Analysis of Multiple Publicly Available Datasets Using Individual Patient Data Approach

**DOI:** 10.1371/journal.pone.0129610

**Published:** 2015-06-16

**Authors:** Nilotpal Chowdhury, Shantanu Sapru

**Affiliations:** 1 Department of Pathology & Laboratory Medicine, All India Institute of Medical Sciences, Rishikesh, Uttarakhand, India; 2 Department of Radiotherapy, All India Institute of Medical Sciences, Rishikesh, Uttarakhand, India; University of Alabama at Birmingham, UNITED STATES

## Abstract

**Introduction:**

Microarray analysis has revolutionized the role of genomic prognostication in breast cancer. However, most studies are single series studies, and suffer from methodological problems. We sought to use a meta-analytic approach in combining multiple publicly available datasets, while correcting for batch effects, to reach a more robust oncogenomic analysis.

**Aim:**

The aim of the present study was to find gene sets associated with distant metastasis free survival (DMFS) in systemically untreated, node-negative breast cancer patients, from publicly available genomic microarray datasets.

**Methods:**

Four microarray series (having 742 patients) were selected after a systematic search and combined. Cox regression for each gene was done for the combined dataset (univariate, as well as multivariate – adjusted for expression of Cell cycle related genes) and for the 4 major molecular subtypes. The centre and microarray batch effects were adjusted by including them as random effects variables. The Cox regression coefficients for each analysis were then ranked and subjected to a Gene Set Enrichment Analysis (GSEA).

**Results:**

Gene sets representing protein translation were independently negatively associated with metastasis in the Luminal A and Luminal B subtypes, but positively associated with metastasis in Basal tumors. Proteinaceous extracellular matrix (ECM) gene set expression was positively associated with metastasis, after adjustment for expression of cell cycle related genes on the combined dataset. Finally, the positive association of the proliferation-related genes with metastases was confirmed.

**Conclusion:**

To the best of our knowledge, the results depicting mixed prognostic significance of protein translation in breast cancer subtypes are being reported for the first time. We attribute this to our study combining multiple series and performing a more robust meta-analytic Cox regression modeling on the combined dataset, thus discovering 'hidden' associations. This methodology seems to yield new and interesting results and may be used as a tool to guide new research.

## Introduction

Microarray analysis has revolutionized our understanding of breast cancer. A molecular classification of breast cancer based on the expression of certain genes has gained acceptance in the last two decades. Luminal A, Luminal B, HER2 positive and Basal subtypes are the major subtypes identified in multiple independent cohorts[1–5], and this classification is being refined further by identification of more subtypes[6–8]. Analyses done on microarrays have suggested that genes related to the cell cycle are of great prognostic importance[9–14].

However, to the best of our knowledge, a comprehensive analysis concentrated on finding prognostically important cellular pathways and processes in the different breast cancer subtypes has not been done. The few studies which have been done have either tried to extract a prognostic gene signature[15–22] or have focused on a small number of pathways[12,23,24]. Furthermore, pathways which are prognostically important independent of the cell cycle related genes have also not been looked at. A significant short coming of existing gene signature studies using microarray technology are methodological flaws related to non-correction of batch effects in microarray data analysis, which may potentially reduce their robustness[25,26].

In order to understand the natural history of breast cancer, it is important to exclude factors that may impact on survival, and which may have changed over the past few decades. We realized that node negative tumors afforded us the best opportunity to study long term survivors and their gene signatures. At present, breast cancer patients are given a variety of treatments, and the responsiveness of these treatments may have an effect on the clinical end point selected, as well as may themselves be associated with gene expression[27–30]. Including patients receiving systemic therapy and those deserving but not receiving any such therapy would increase the heterogeneity of the analysis, thus rendering any modeling non-robust, and the results difficult to interpret. This problem is compounded further by the non-availability of the exact treatment regimen in most genomics data sets. The possibility of different treatment regimens being associated in a variable, non-uniform manner with gene expression renders the task of appropriate modeling and interpretation of results even more difficult. In order to exclude such treatment related effects, we selected only systemically untreated patients for our study. This would have the advantage of studying the true tumor related natural history un-confounded by treatment related effects. We included only those microarray data sets from which the batch in which the analysis was done could be deduced, so that the batch effects could be controlled for while analyzing the data.

The present study focused on finding significant pathways and processes associated with distant metastasis in node negative breast cancer. We tried finding not only the main processes associated with distant metastasis in the entire dataset, but also those which were significant independent of cell cycle. Analysis was also carried out on the different molecular subtypes separately.

## Methods

### Data sources and study selection

**Fig 1:**
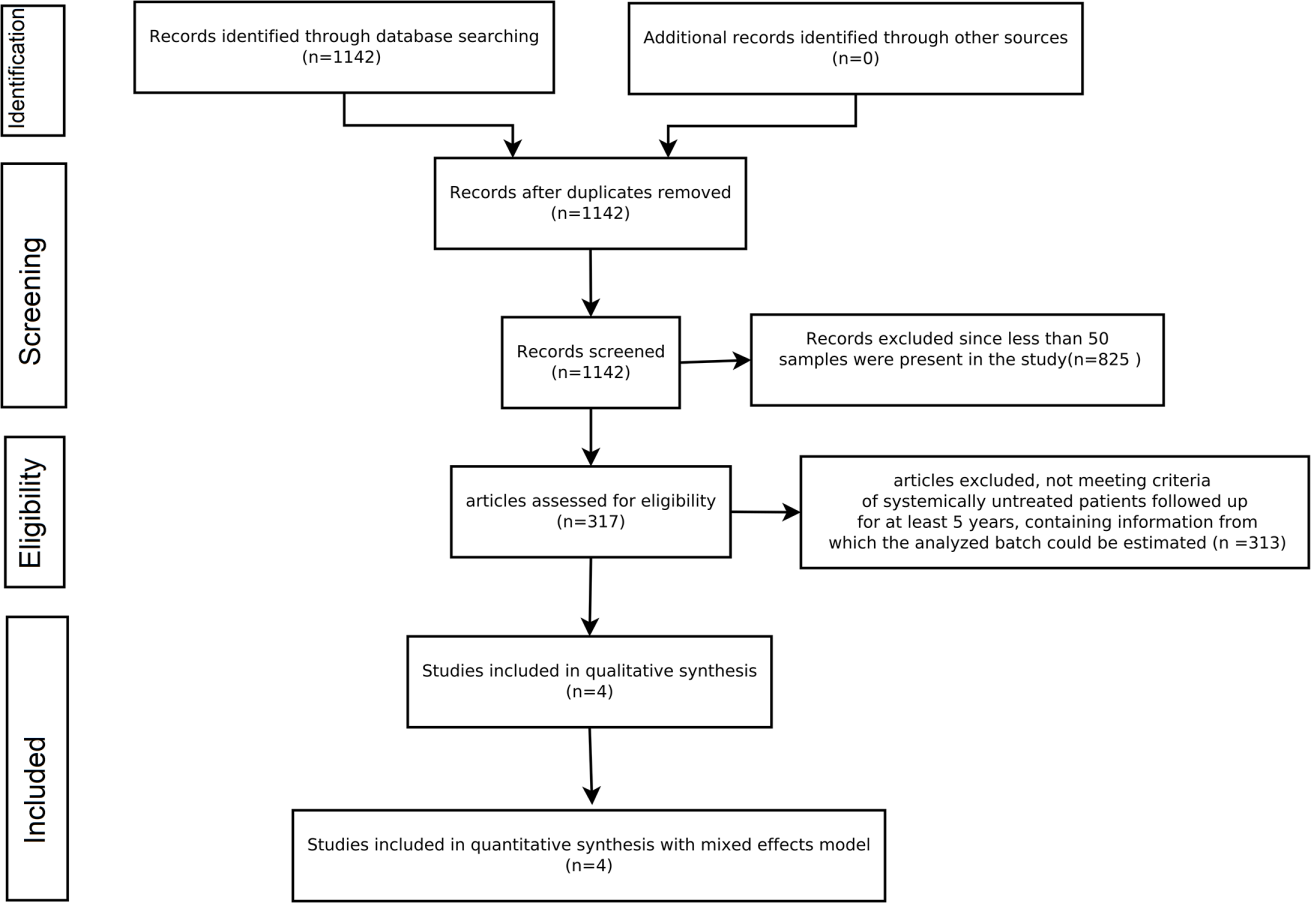
The PRISMA Flow diagram showing the method behind the inclusion of studies for the analysis.

The Gene Expression Omnibus (GEO) database was queried in June 2014 for node negative breast cancer using the search terms “breast cancer” and “breast neoplasms”, combining them with Boolean operator "OR", filtered to include only Gene Expression Series (“gse”), for organism “Homo sapiens” containing “Expression profiling by array”. An initial search resulted in 1142 series. From the 1142 series, only those having 50 or more samples were included. This resulted in 317 data series. Of these 317 data series, only 5 contained arrays exclusively from node-negative breast cancer patients without any systemic adjuvant therapy. The rest 312 were excluded because they did not meet our inclusion criteria of node negative patients not receiving any systemic adjuvant therapy and having follow up data of a minimum of 05 years. Of the five remaining series, one did not contain information about the batch or date in which the microarrays were analyzed, and was excluded. Finally we were left with four series, viz. GSE2034[31], GSE5327[32], GSE7390[33], and GSE11121[34], all having confirmed node negative patients who did not receive any form of systemic therapy. The flow chart summarizing the steps in study selection is given in Fig 1.

### Data extraction and synthesis

The raw microarray data from each series was downloaded and pre-processed separately, as recommended by Hahne et al [35]using the frozen Robust Multi-array Average (fRMA) algorithm[36]. The different series were then combined together with respect to our primary end point of interest Distant Metastasis Free Survival (DMFS) or distant recurrence, depending on coding of particular data series. The occurrence of distant metastasis was taken as the event of interest for which time-to-event analyses were done. The series were censored at 10 years after diagnosis.

Prior to using a Cox regression model, the probes were collapsed to genes, by the WGCNA package[37]. Probes which gave the maximum average expression were selected to represent the gene.

Only the four major molecular sub-types were investigated using the PAM50 classifier[5], i.e. Basal, HER2 Positive, Luminal A and Luminal B subtypes.

Six different analyses were conducted combining data from the four studies as follows: (a) Univariate analysis on the entire combined dataset, (b) Multivariate analysis on the combined dataset adjusting for expression of cell cycle related genes, (c) Univariate analysis on the HER2 subtype extracted from the combined dataset, (d) Univariate analysis on the basal subtype extracted from the combined dataset, (e) Univariate analysis on the Luminal A subtype extracted from the combined dataset, and, (f) Univariate analysis on the Luminal B subtype extracted from the combined dataset.

To adjust for expression of cell cycle related genes for the multivariate analysis done in analysis (b) listed above, the AURKA module score[12] as implemented in the *genefu* package in Bioconductor and R was added to the Cox regression model.

Each analysis (a-f above), was evaluated by Cox regression models which included the gene expression as a fixed effects variable and one of the following as a random effects variable: i) Batch (as estimated from the scan date of the original microarray analysis), ii) Study series, iii) Centre or institution, iv) Batch as a nested effect in the Series, and, v) Batch as a nested effect in the Centre.

A sixth model (vi) was also performed as a control which did not include the batch or centre (a simple marginal model with the gene expression as the sole variable).

Therefore, in each analysis (a-f above), for each gene, six different Cox regression models were evaluated. For each analysis, the model which gave the lowest median Akaike Information Criterion (AIC)[38] value was selected as the most appropriate model for that particular analysis.

The coefficients obtained from the most appropriate Cox regression models (for each analysis) were then ranked according to their value. Since the standard GSEA (Gene Set Enrichment Analysis) currently lacks the software implementation for adjusting for batch effects, as well as being incapable of performing time-to event analysis, hence the coefficients obtained were subjected to a pre-ranked GSEA[39,40].

The following downloaded gene set collections were used in the present study: i) The gene sets representing the Canonical pathways (presented as C2: CP gene set collections), which included gene sets from the Biocarta[41], KEGG,[42,43] Reactome[44] and Protein Interaction Database[45] repositories, and, ii) The Gene Ontology[46,47] gene sets (presented as C5 gene set collections).

A Family Wise Error Rate (FWER)-adjusted p-value of ≤0.05 was selected as our level of significance for the pre-ranked GSEA. Ten thousand permutations were used (default being one thousand); the default setting was kept for all the other parameters of the GSEA study. The gene sets which were found significant in the pre-ranked GSEA analysis were then extracted.

**Fig 2:**
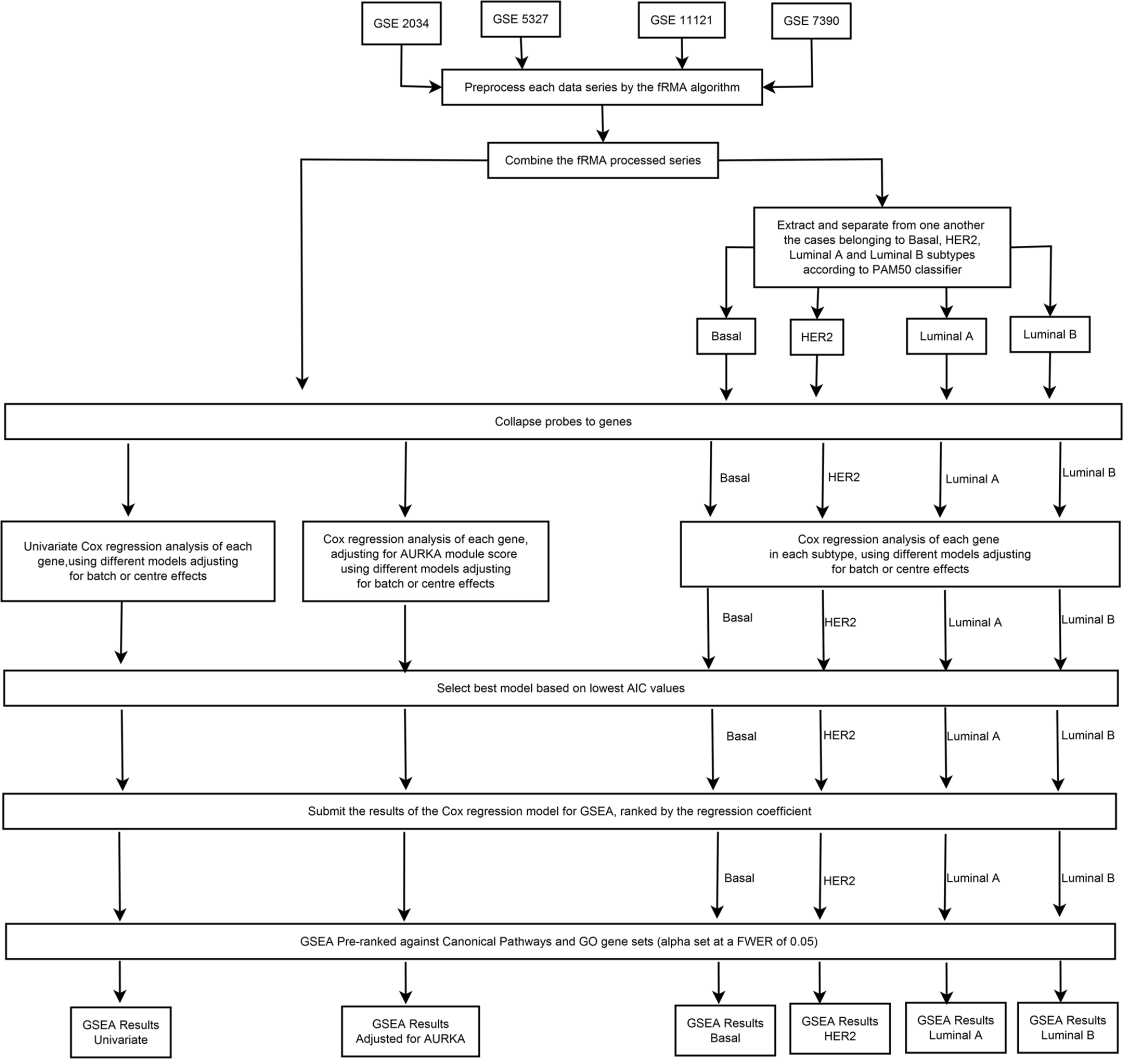
Flow chart of present analysis till the Pre-ranked GSEA to find DMFS associated gene sets.

The entire workflow is described in Fig 2.

Visualization of the relationship between the different significant gene sets from the pre-ranked GSEA above was done by means of the Enrichment Map Cytoscape plugin[48], which groups the highly redundant gene sets in clusters. The Overlap coefficient[48], at a threshold of 0.5, was used for clustering the gene sets, as recommended in the Enrichment Map manual.

The "leading edges" of significant gene sets from each of the six analyses (a-f) were then studied after the pre-ranked GSEA. Genes which were the most commonly distributed among the leading edges of the significant gene sets were identified for each analysis.

A standard GSEA was performed again at the end to find the relationship between each gene set included in each analysis (a-f above) and the AURKA module score for each data series. For this analysis, the “Pearson” metric for ranking genes was used, as recommended by the GSEA user manual; no correction was made for the batch effects, and one thousand permutations were used in the analysis of each data series. A final adjusted p-value was calculated by combining the p-values obtained from the four studies by the Stouffers method[49]. The FWER was calculated by the method of Holm[50]. An FWER-adjusted p-value of ≤0.05 was selected as our level of significance.

Microarray preprocessing, categorization into subtypes and Cox regression were carried out by the statistical environment R v3.1.0[51] using the coxme[52], GEOquery[53], affy[54], genefu[55], hgu133a.db[56] and WGCNA[37] packages.

All the gene sets used in the GSEA were downloaded from the website of the Broad institute[57]:

The GSEA analyses were carried out by GSEA2-2.0.14 software downloaded from the Broad institute.

## Results

The Clinical characteristics of the patients in the data series are given in Table 1, while the tumor-related characteristics are detailed in Table 2.

**Table 1:** Summary of patient characteristics of the four series analysed.

		GSE2034(N = 286)	GSE5327(N = 58)	GSE11121(N = 200)	GSE7390(N = 198)
**Median Follow up period in years (range)**		8.8 (0.17–14.25)	7.4 (0.36–13.04)	8.6 (0.08–20.00)	13.7 (0.34–24.95)
**Mean patient age in years (range)**		54 (26–83)	Unknown	60 (25–90)	46 (24–60)
**Radiotherapy given**	Yes	248	—	125	—
	No	38	—	75	—
	Unknown	00	58	00	198

**Table 2:** Summary of tumor characteristics of the four series analysed.

		GSE2034	GSE5327	GSE11121	GSE7390
**Tumor size**	T1	146 (51%)	—	111 (56%)	102 (52%)
	T2	132 (46%)	—	81 (40%)	96 (48%)
	T3/4	8 (3%)	—	8 (4%)	0 (0%)
	Unknown	0 (0%)	58 (100%)	0 (0%)	0 (0%)
**Tumor Grade**	Well differentiated	7 (2%)	—	41 (21%)	30 (15%)
	Moderately	42 (15%)	—	110 (55%)	83 (42%)
	Poorly differentiated	148 (52%)	—	45 (23%)	83 (42%)
	Unknown	89 (31%)	58 (100%)	4 (2%)	2 (1%)
**Molecular subtypes**	Basal	58 (20%)	38 (66%)	22 (11%)	45 (23%)
	HER2	41 (14%)	12 (21%)	21(11%)	26(13%)
	Luminal A	108 (38%)	4 (7%)	104 (52%)	71 (36%)
	Luminal B	62 (22%)	2 (3%)	46 (23%)	46 (23%)
	Normal-like	17 (6%)	32(3%)	7 (4%)	10(5%)
**ER**	Negative	77 (27%)	58 (100%)	44 (22%)	64 (32%)
	Positive	209 (73%)	0 (0%)	156 (78%)	134 (68%)
	Unknown	0 (0%)	0 (0%)	0 (0%)	0 (0%)
**PR**	Negative	111 (39%)	—	70 (35%)	—
	Positive	165 (58%)	—	130 (65%)	—
	Unknown	10 (3%)	58 (100%)	0 (0%)	198 (100%)

The comparability of the expression values from the different samples was visualized using boxplots. fRMA pre-processed expression data were seen to be comparable between samples, with the distribution of the expression values between different arrays being similar to one another (S1 Fig). This supported the contention that the fRMA pre-processed data from different series could be combined together for analysis.

Of the various mixed models considered, the model with just batch effects as random variable (Cox regression model (i) in Methods above) gave the lowest AIC values for the analyses from (a)-(d) and analysis (f) above. For analysis (e), i.e. Univariate analysis on the Luminal A subtype, the cox regression model with Batch nested in Centre as RE Variable (model (v) in methods above) gave the lowest AIC values. These models (which gave the lowest AIC value for an analysis) were selected as the most appropriate model for that particular analysis.

Details of the AIC values are shown in S1 Table.

**Note on interpretation of the GSEA results**: GSEA is a competitive gene set analysis method. In this context, a gene set negatively associated with metastasis means that it is more negatively associated with metastasis than random gene sets formed from genes other than those in the gene set tested. A gene set positively associated with metastasis means that it is more positively associated with metastasis than random gene sets formed from genes other than those in the gene set tested.

### I) Results of the analysis on the entire combined dataset

**Fig 3:**
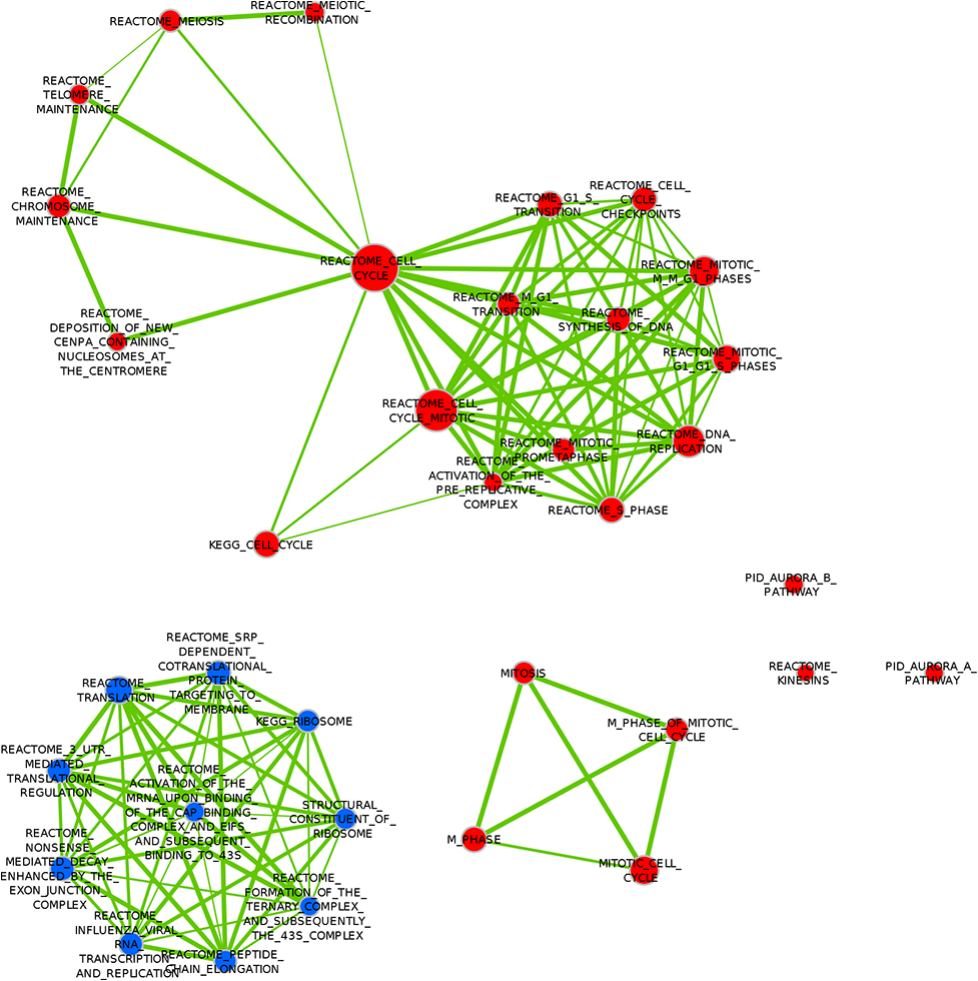
Enrichment Map visualization of DMFS-associated gene sets on Univariate analysis in the Combined Dataset. Red nodes represent gene sets positively associated with metastasis. Blue nodes represent gene sets negatively associated with metastasis. Two nodes are connected by edges (solid green lines) only if the overlap coefficient between the nodes is greater than 0.5, and the edge thickness increases with increased Overlap coefficient.

When the combined dataset was analyzed in a univariate setting (analysis (a) in Methods above), gene sets related to protein translation were found to be negatively associated with distant metastasis (good prognosis), as depicted in Table 3 and Fig 3; while gene sets related to the Cell cycle (i.e. proliferation-related gene sets) were the only gene sets to be significantly positively associated with metastasis (poor prognosis) (Table 4, Fig 3).

**Table 3:** Gene sets negatively associated with distant metastasis (good prognosis) on univariate analysis of the combined dataset.

Name of gene set	Pathway/Term to which the gene set belongs or is a subset of	Name of curating database	ES	NES	p-val	FWER
REACTOME_PEPTIDE_CHAIN_ELONGATION	Gene Expression; Translation; Eukaryotic translation elongation;	Reactome	-0.662	-2.761	<0.0001	<0.0001
KEGG_RIBOSOME	Genetic Information Processing; Translation;	KEGG	-0.654	-2.747	<0.0001	<0.0001
REACTOME_3_UTR_MEDIATED_TRANSLATIONAL_REGULATION	3” UTR Mediated Translational regulation	Reactome	-0.623	-2.736	<0.0001	<0.0001
REACTOME_TRANSLATION	Gene Expression; Translation	Reactome	-0.561	-2.612	<0.0001	<0.0001
STRUCTURAL_CONSTITUENT_OF_RIBOSOME	Molecular function; Structural Molecular activity	Gene Ontology	-0.609	-2.526	<0.0001	<0.0001
REACTOME_SRP_DEPENDENT_COTRANSLATIONAL_PROTEIN_TARGETING_TO_MEMBRANE	Gene Expression; Translation	Reactome	-0.569	-2.506	<0.0001	<0.0001
REACTOME_FORMATION_OF_THE_TERNARY_COMPLEX_AND_SUBSEQUENTLY_THE_43S_COMPLEX	Gene Expression; Translation; Eukaryotic translation initiation; CAP dependent translation initiation;	Reactome	-0.649	-2.449	<0.0001	0.0001
REACTOME_NONSENSE_MEDIATED_DECAY_ENHANCED_BY_THE_EXON_JUNCTION_COMPLEX	Gene Expression; Nonsense mediated decay	Reactome	-0.548	-2.411	<0.0001	0.0003
REACTOME_ACTIVATION_OF_THE_MRNA_UPON_BINDING_OF_THE_CAP_BINDING_COMPLEX_AND_EIFS_AND_SUBSEQUENT_BINDING_TO_43S	Gene Expression; Translation; Eukaryotic translation initiation; CAP dependent translation initiation	Reactome	-0.618	-2.41	<0.0001	0.0005
REACTOME_INFLUENZA_VIRAL_RNA_TRANSCRIPTION_AND_REPLICATION	Disease; Influenza Infection; Influenza Life Cycle	Reactome	-0.537	-2.354	<0.0001	0.0016

(ES = Enrichment Score, NES = Normalized Enrichment Score, p-val = unadjusted p-value, FWER = Family Wise Error Rate-adjusted p-value)

**Table 4:** Gene sets positively associated with distant metastasis (bad prognosis) on univariate analysis of the combined dataset.

Name of gene set	Pathway/Term to which the gene set belongs or is a subset of	Name of curating database	ES	NES	p-val	FWER
REACTOME_DNA_REPLICATION	DNA Replication	Reactome	0.570	2.332	<0.0001	<0.0001
REACTOME_MITOTIC_M_M_G1_PHASES	Cell cycle	Reactome	0.573	2.315	<0.0001	<0.0001
REACTOME_CELL_CYCLE	Cell cycle	Reactome	0.520	2.250	<0.0001	<0.0001
REACTOME_CHROMOSOME_MAINTENANCE	Cell cycle	Reactome	0.598	2.244	<0.0001	<0.0001
REACTOME_CELL_CYCLE_MITOTIC	Cell cycle	Reactome	0.511	2.187	<0.0001	0.0008
REACTOME_G1_S_TRANSITION	Cell cycle	Reactome	0.565	2.167	<0.0001	0.0011
M_PHASE_OF_MITOTIC_CELL_CYCLE	Cell cycle; Cell Cycle, Mitotic	Reactome	0.582	2.131	<0.0001	0.0031
REACTOME_KINESINS	Hemostasis	Reactome	0.777	2.126	<0.0001	0.0035
REACTOME_S_PHASE	Cell cycle; Cell Cycle Mitotic	Reactome	0.552	2.117	<0.0001	0.0046
REACTOME_DEPOSITION_OF_NEW_CENPA_CONTAINING_NUCLEOSOMES_AT_THE_CENTROMERE	Cell cycle; Chromosome maintenance; Nucleosome Assembly	Reactome	0.675	2.116	<0.0001	0.0046
REACTOME_SYNTHESIS_OF_DNA	Cell cycle; Cell Cycle, Mitotic; S Phase; DNA Replication	Reactome	0.563	2.105	<0.0001	0.0060
PID_AURORA_A_PATHWAY	Cell cycle pathways, mitotic	PID	0.684	2.103	<0.0001	0.0063
REACTOME_MITOTIC_PROMETAPHASE	Cell cycle; Cell Cycle, Mitotic; M Phase	Reactome	0.575	2.098	<0.0001	0.0067
REACTOME_M_G1_TRANSITION	Cell cycle; Cell Cycle, Mitotic	Reactome	0.575	2.092	<0.0001	0.0073
REACTOME_CELL_CYCLE_CHECKPOINTS	Cell cycle	Reactome	0.546	2.088	<0.0001	0.0088
REACTOME_MITOTIC_G1_G1_S_PHASES	Cell cycle; Cell Cycle Mitotic	Reactome	0.530	2.086	<0.0001	0.0093
REACTOME_MEIOSIS	Cell cycle	Reactome	0.579	2.075	<0.0001	0.0124
MITOSIS	Biological Process; Cell Cycle; Mitotic Cell Cycle	Gene Ontology	0.570	2.065	<0.0001	0.0165
M_PHASE	Biological Process; Cell Cycle Phase	Gene Ontology	0.537	2.056	<0.0001	0.0193
KEGG_CELL_CYCLE	Cellular Processes; Cell growth and death	KEGG	0.527	2.056	<0.0001	0.0193
PID_AURORA_B_PATHWAY	Cell cycle pathways, mitotic	PID	0.643	2.049	<0.0001	0.0220
REACTOME_ACTIVATION_OF_THE_PRE_REPLICATIVE_COMPLEX	Cell cycle; Cell Cycle, Mitotic; M/G1 Transition; DNA Replication Pre-initiation; DNA Replication	Reactome	0.671	2.045	0.0001	0.0235
MITOTIC_CELL_CYCLE	Biological Process; Cell Cycle	Gene Ontology	0.512	2.044	<0.0001	0.0241
REACTOME_TELOMERE_MAINTENANCE	Cell cycle; Chromosome maintenance	Reactome	0.600	2.035	<0.0001	0.0292
REACTOME_MEIOTIC_RECOMBINATION	Cell cycle; Meiosis	Reactome	0.617	2.016	<0.0001	0.0427

(ES = Enrichment Score, NES = Normalized Enrichment Score, p-val = unadjusted p-value, FWER = Family Wise Error Rate-adjusted p-value)

On performing the multivariate analysis adjusted for the Cell cycle by adding the AURKA module to the Cox regression model (analysis (b)), gene sets representing the integrin1 pathway and the proteinaceous extracellular matrix were positively associated with metastasis (Table 5).

**Table 5:** Gene sets positively associated with distant metastasis (bad prognosis) after adjustment for proliferation using the AURKA module score in the combined dataset.

Name of gene set	Pathway/Term to which the gene set belongs or is a subset of	Name of curating database	ES	NES	p-val	FWER
PID_INTEGRIN1_PATHWAY	Beta 1 integrin cel surface interactions	Protein Interaction Database	0.573	2.105	<0.001	0.0163
PROTEINACEOUS_EXTRACELLULAR_MATRIX	Cellular component; Extracellular region; Extracellular region part;Extracellular matrix	Gene Ontology	0.528	2.069	<0.001	0.0335

(ES = Enrichment Score, NES = Normalized Enrichment Score, p-val = unadjusted p-value, FWER = Family Wise Error Rate-adjusted p-value)

### II) Results of the subtype analysis

The results for the gene sets significantly associated with distant metastasis in the individual molecular subtypes are detailed below.

#### Univariate analysis on HER2 tumors (Analysis (c) in Methods)

Gene sets related to interferon gamma signaling was the only gene set found negatively associated with distant metastasis (Table 6). No gene sets were found to be positively correlated with distant metastasis.

**Table 6:** Gene sets negatively associated with distant metastasis (good prognosis) in the different subtypes of breast cancer.

Subtype	Name of gene set	Pathway/Term to which the gene set belongs or is a subset of	Name of curating database	ES	NES	p-val	FWER
HER2	REACTOME_INTERFERON_GAMMA_SIGNALING	Immune System; Cytokine signalling in immune system; Interferon signalling	Reactome	-0.575	-2.15	<0.0001	0.0414
Luminal B	REACTOME_PEPTIDE_CHAIN_ELONGATION	Gene Expression; Translation; Eukaryotic translation elongation	Reactome	-0.751	-2.688	<0.0001	<0.0001
	KEGG_RIBOSOME	Genetic Information Processing; Translation	KEGG	-0.742	-2.669	<0.0001	<0.0001
	REACTOME_3_UTR_MEDIATED_TRANSLATIONAL_REGULATION	3” UTR Mediated Translational regulation	Reactome	-0.690	-2.563	<0.0001	<0.0001
	STRUCTURAL_CONSTITUENT_OF_RIBOSOME	Molecular function; Structural Molecular activity	Gene Ontology	-0.720	-2.537	<0.0001	<0.0001
	REACTOME_NONSENSE_MEDIATED_DECAY_ENHANCED_BY_THE_EXON_JUNCTION_COMPLEX	Gene Expression; Nonsense mediated decay	Reactome	-0.640	-2.394	<0.0001	<0.0001
	REACTOME_SRP_DEPENDENT_COTRANSLATIONAL_PROTEIN_TARGETING_TO_MEMBRANE	Gene Expression; Translation	Reactome	-0.610	-2.293	<0.0001	0.0001
	REACTOME_TRANSLATION	Gene Expression; Translation	Reactome	-0.576	-2.271	<0.0001	0.0001
	REACTOME_INFLUENZA_VIRAL_RNA_TRANSCRIPTION_AND_REPLICATION	Disease; Influenza Infection; Influenza Life Cycle	Reactome	-0.608	-2.263	<0.0001	0.0003
	REACTOME_FORMATION_OF_THE_TERNARY_COMPLEX_AND_SUBSEQUENTLY_THE_43S_COMPLEX	Gene Expression; Translation; Eukaryotic translation initiation; CAP dependent translation initiation;	Reactome	-0.689	-2.243	<0.0001	0.0003
	REACTOME_ACTIVATION_OF_THE_MRNA_UPON_BINDING_OF_THE_CAP_BINDING_COMPLEX_AND_EIFS_AND_SUBSEQUENT_BINDING_TO_43S	Gene Expression; Translation; Eukaryotic translation initiation; CAP dependent translation initiation	Reactome	-0.665	-2.220	<0.0001	0.0009
Luminal A	REACTOME_PEPTIDE_CHAIN_ELONGATION	Gene Expression; Translation; Eukaryotic translation elongation	Reactome	-0.712	-2.757	<0.0001	<0.0001
	REACTOME_3_UTR_MEDIATED_TRANSLATIONAL_REGULATION	3” UTR Mediated Translational regulation	Reactome	-0.674	-2.716	<0.0001	<0.0001
	STRUCTURAL_CONSTITUENT_OF_RIBOSOME	Molecular function; Structural Molecular activity	Gene Ontology	-0.704	-2.672	<0.0001	<0.0001
	KEGG_RIBOSOME	Genetic Information Processing; Translation	KEGG	-0.682	-2.635	<0.0001	<0.0001
	REACTOME_INFLUENZA_VIRAL_RNA_TRANSCRIPTION_AND_REPLICATION	Disease; Influenza Infection; Influenza Life Cycle	Reactome	-0.634	-2.537	<0.0001	<0.0001
	REACTOME_NONSENSE_MEDIATED_DECAY_ENHANCED_BY_THE_EXON_JUNCTION_COMPLEX	Gene Expression; Nonsense mediated decay	Reactome	-0.622	-2.506	<0.0001	<0.0001
	REACTOME_TRANSLATION	Gene Expression; Translation	Reactome	-0.557	-2.366	<0.0001	0.0003
	REACTOME_SRP_DEPENDENT_COTRANSLATIONAL_PROTEIN_TARGETING_TO_MEMBRANE	Gene Expression; Translation	Reactome	-0.556	-2.247	<0.0001	0.0028
	REACTOME_FORMATION_OF_THE_TERNARY_COMPLEX_AND_SUBSEQUENTLY_THE_43S_COMPLEX	Gene Expression; Translation; Eukaryotic translation initiation; CAP dependent translation initiation;	Reactome	-0.611	-2.139	<0.0001	0.0202
	REACTOME_ACTIVATION_OF_THE_MRNA_UPON_BINDING_OF_THE_CAP_BINDING_COMPLEX_AND_EIFS_AND_SUBSEQUENT_BINDING_TO_43S	Gene Expression; Translation; Eukaryotic translation initiation; CAP dependent translation initiation	Reactome	-0.595	-2.133	<0.0001	0.0227

(ES = Enrichment Score, NES = Normalized Enrichment Score, p-val = unadjusted p-value, FWER = Family Wise Error Rate-adjusted p-value)

#### Univariate analysis on Basal tumors (Analysis (d))

**Fig 4:**
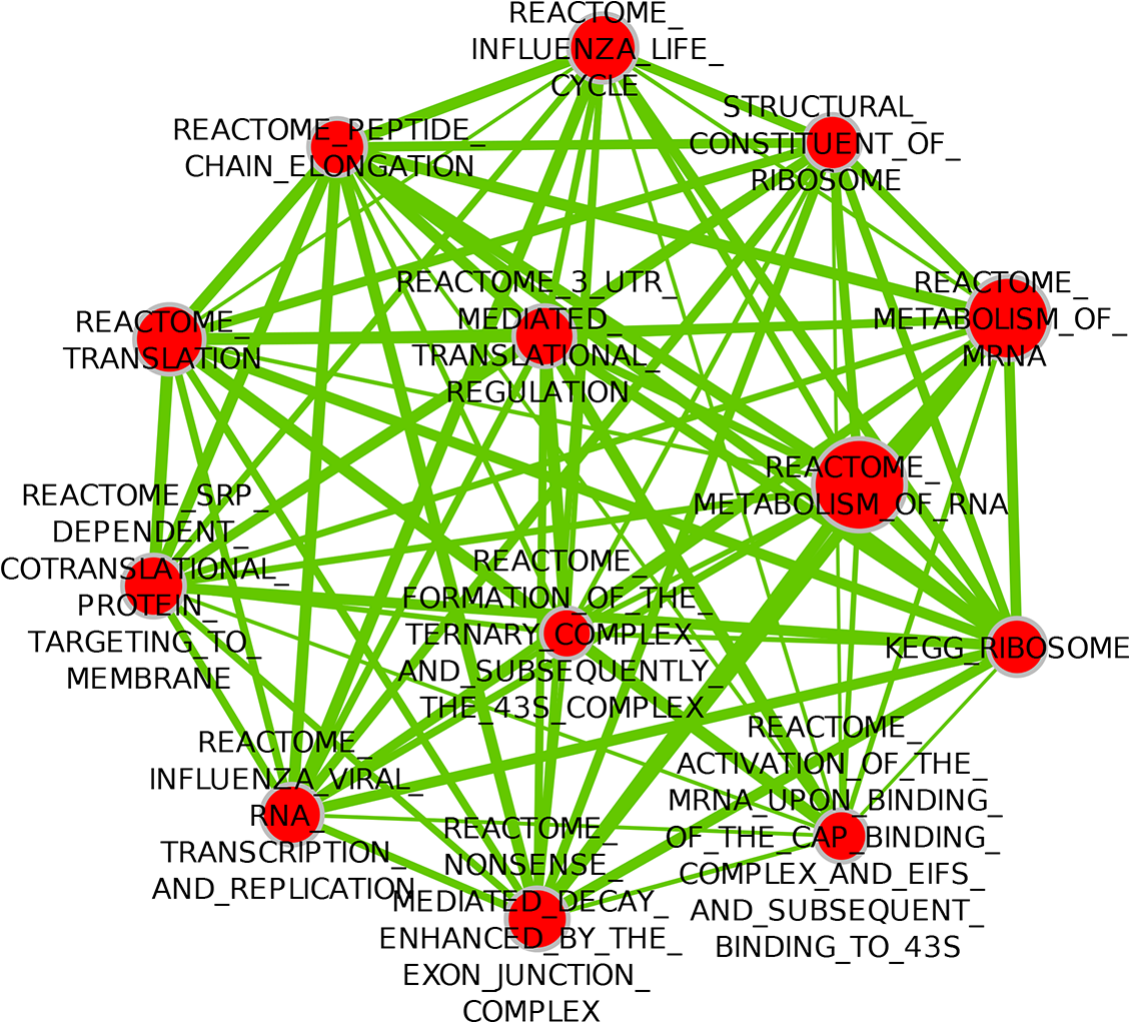
Enrichment Map visualization of DMFS-associated gene sets in the Basal Subtype. Red nodes represent gene sets positively associated with metastasis. Blue nodes represent gene sets negatively associated with metastasis (none present in this figure). Two nodes are connected by edges (solid green lines) only if the overlap coefficient between the nodes is greater than 0.5, and the edge thickness increases with increased Overlap coefficient.

Gene sets representing protein translation were the only sets significantly positively associated with metastasis (Table 7, Fig 4).

**Table 7:** Gene Sets positively associated with distant metastasis (bad prognosis) in the different subtypes of breast cancer.

Subtype	Name of gene set	Pathway/Term to which the gene set belongs or is a subset of	Name of curating database	ES	NES	p-val	FWER
Basal	REACTOME_PEPTIDE_CHAIN_ELONGATION	Gene Expression; Translation; Eukaryotic translation elongation	Reactome	0.828	3.073	<0.0001	<0.0001
	REACTOME_3_UTR_MEDIATED_TRANSLATIONAL_REGULATION	3” UTR Mediated Translational regulation	Reactome	0.795	3.066	<0.0001	<0.0001
	KEGG_RIBOSOME	Genetic Information Processing; Translation	KEGG	0.819	3.038	<0.0001	<0.0001
	REACTOME_INFLUENZA_VIRAL_RNA_TRANSCRIPTION_AND_REPLICATION	Disease; Influenza Infection; Influenza Life Cycle	Reactome	0.770	2.965	<0.0001	<0.0001
	REACTOME_NONSENSE_MEDIATED_DECAY_ENHANCED_BY_THE_EXON_JUNCTION_COMPLEX	Gene Expression; Nonsense mediated decay	Reactome	0.752	2.938	<0.0001	<0.0001
	STRUCTURAL_CONSTITUENT_OF_RIBOSOME	Molecular function; Structural Molecular activity	Gene Ontology	0.795	2.907	<0.0001	<0.0001
	REACTOME_SRP_DEPENDENT_COTRANSLATIONAL_PROTEIN_TARGETING_TO_MEMBRANE	Gene Expression; Translation	Reactome	0.734	2.858	<0.0001	<0.0001
	REACTOME_TRANSLATION	Gene Expression; Translation	Reactome	0.688	2.820	<0.0001	<0.0001
	REACTOME_INFLUENZA_LIFE_CYCLE	Disease; Influenza Infection;	Reactome	0.693	2.812	<0.0001	<0.0001
	REACTOME_ACTIVATION_OF_THE_MRNA_UPON_BINDING_OF_THE_CAP_BINDING_COMPLEX_AND_EIFS_AND_SUBSEQUENT_BINDING_TO_43S	Gene Expression; Translation; Eukaryotic translation initiation; CAP dependent translation initiation	Reactome	0.729	2.512	<0.0001	<0.0001
	REACTOME_FORMATION_OF_THE_TERNARY_COMPLEX_AND_SUBSEQUENTLY_THE_43S_COMPLEX	Gene Expression; Translation; Eukaryotic translation initiation; CAP dependent translation initiation;	Reactome	0.739	2.476	<0.0001	0.0001
	REACTOME_METABOLISM_OF_MRNA	Metabolism of mRNA	Reactome	0.546	2.366	<0.0001	0.0002
	REACTOME_METABOLISM_OF_RNA		Reactome	0.499	2.217	<0.0001	0.0030
Luminal B	REACTOME_DNA_REPLICATION	DNA Replication	Reactome	0.486	2.140	<0.0001	0.0115
	REACTOME_G1_S_TRANSITION	Cell cycle	Reactome	0.519	2.128	<0.0001	0.0135
	REACTOME_CELL_CYCLE_MITOTIC	Cell cycle	Reactome	0.448	2.082	<0.0001	0.0339
	REACTOME_S_PHASE	Cell cycle; Cell Cycle Mitotic	Reactome	0.510	2.068	<0.0001	0.0417
Luminal A	REACTOME_KINESINS	Hemostasis	Reactome	0.800	2.073	<0.0001	0.0096

(ES = Enrichment Score, NES = Normalized Enrichment Score, p-val = unadjusted p-value, FWER = Family Wise Error Rate-adjusted p-value)

#### Univariate analysis on Luminal A tumors (Analysis (e))

**Fig 5:**
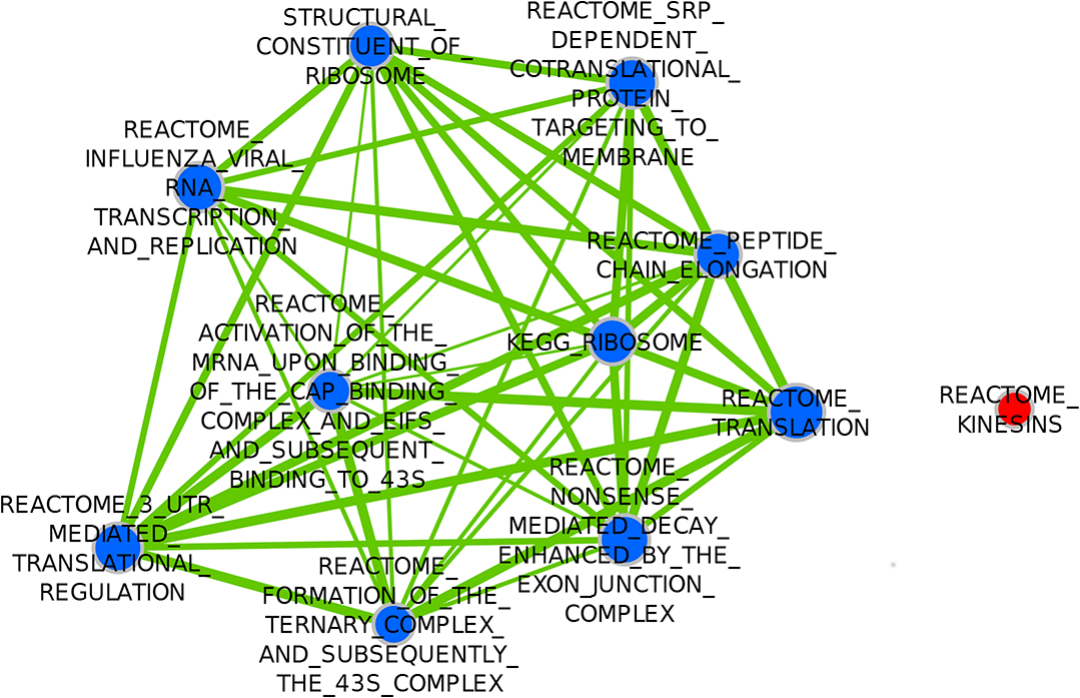
Enrichment Map visualization of DMFS-associated gene sets in the Luminal A Subtype.

Gene sets representing translation were negatively associated with distant metastasis (Table 6, Fig 5). One gene set representing Kinesins (thus possibly related to mitosis)were significantly positively associated with metastasis (i.e. negatively associated with survival) (Table 7, Fig 5)

#### Univariate analysis on Luminal B tumors (Analysis (f))

**Fig 6:**
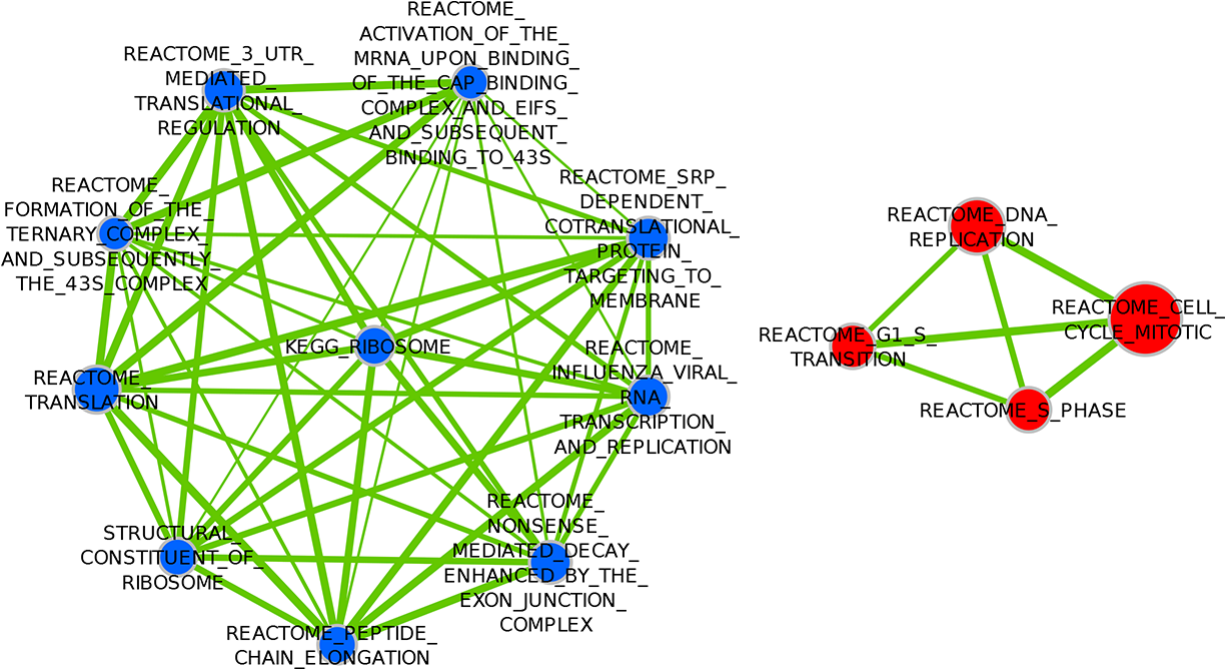
Enrichment Map visualization of DMFS-associated gene sets in the Luminal B Subtype. Red nodes represent gene sets positively associated with metastasis. Blue nodes represent gene sets negatively associated with metastasis. Two nodes are connected by edges (solid green lines) only if the overlap coefficient between the nodes is greater than 0.5, and the edge thickness increases with increased Overlap coefficient.

Gene sets representing translation were negatively associated with distant metastasis (Table 6, Fig 6). Gene sets associated with cell cycle (proliferation) dominated the sets significantly positively associated with metastasis (i.e. negatively associated with survival) (Table 7, Fig 6).

### III) Results of the leading edge analysis

#### Univariate analysis on the entire combined dataset

Genes encoding for various ribosomal proteins were commonly distributed among the leading edges of the gene sets significantly associated with good prognosis, while genes encoding for replication proteins A1 and A3, DNA directed polymerase and Cyclin dependent Kinase 2 were commonly distributed among the leading edges of the significant gene sets associated with bad prognosis (Tables 8 and 9).

**Table 8:** Genes which are found in a large number of leading edges of significant gene sets negatively associated with distant metastasis (good prognosis) for each analysis.

Analysis	Entrez Gene ID	Symbol	Gene name	Cytogenetic location	Proportion of the leading edges the gene is found
Univariate	6191	RPS4X	ribosomal protein S4, X-linked	Xq13.1	10/10
	6193	RPS5	ribosomal protein S5	19q13.4	10/10
	6194	RPS6	ribosomal protein S6	9p21	10/10
	6202	RPS8	ribosomal protein S8	1p34.1-p32	10/10
	6203	RPS9	ribosomal protein S9	19q13.4	10/10
	6209	RPS15	ribosomal protein S15	19p13.3	10/10
	6210	RPS15A	ribosomal protein S15a	16p	10/10
	6217	RPS16	ribosomal protein S16	19q13.1	10/10
	6223	RPS19	ribosomal protein S19	19q13.2	10/10
	6229	RPS24	ribosomal protein S24	10q22	10/10
	6230	RPS25	ribosomal protein S25	11q23.3	10/10
	6234	RPS28	ribosomal protein S28	19p13.2	10/10
Luminal A	6191	RPS4X	ribosomal protein S4, X-linked	Xq13.1	10/10
	6193	RPS5	ribosomal protein S5	19q13.4	10/10
	6194	RPS6	ribosomal protein S6	9p21	10/10
	6203	RPS9	ribosomal protein S9	19q13.4	10/10
	6209	RPS15	ribosomal protein S15	19p13.3	10/10
	6217	RPS16	ribosomal protein S16	19q13.1	10/10
	6223	RPS19	ribosomal protein S19	19q13.2	10/10
	6229	RPS24	ribosomal protein S24	10q22	10/10
	6230	RPS25	ribosomal protein S25	11q23.3	10/10
	6234	RPS28	ribosomal protein S28	19p13.2	10/10
	6235	RPS29	ribosomal protein S29	14q	10/10
Luminal B	2197	FAU	Finkel-Biskis-Reilly murine sarcoma virus (FBR-MuSV) ubiquitously expressed	11q13	10/10
	6188	RPS3	ribosomal protein S3	11q13.3-q13.5	10/10
	6191	RPS4X	ribosomal protein S4, X-linked	Xq13.1	10/10
	6192	RPS4Y1	ribosomal protein S4, Y-linked 1	Yp11.3	10/10
	6194	RPS6	ribosomal protein S6	9p21	10/10
	6201	RPS7	ribosomal protein S7	2p25	10/10
	6202	RPS8	ribosomal protein S8	1p34.1-p32	10/10
	6203	RPS9	ribosomal protein S9	19q13.4	10/10
	6206	RPS12	ribosomal protein S12	6q23.2	10/10
	6209	RPS15	ribosomal protein S15	19p13.3	10/10
	6210	RPS15A	ribosomal protein S15a	16p	10/10
	6222	RPS18	ribosomal protein S18	6p21.3	10/10
	6223	RPS19	ribosomal protein S19	19q13.2	10/10
	6229	RPS24	ribosomal protein S24	10q22	10/10
	6230	RPS25	ribosomal protein S25	11q23.3	10/10
	6234	RPS28	ribosomal protein S28	19p13.2	10/10

**Table 9:** Genes which are found in a large number of leading edges of significant gene sets positively associated with distant metastasis (bad prognosis) for each analysis.

Analysis	Entrez Gene ID	Symbol	Gene name	Cytogenetic location	Proportion of the leading edges the gene is found
Univariate	1017	CDK2	cyclin-dependent kinase 2	12q13	15/25
	6117	RPA1	replication protein A1, 70kDa	17p13.3	15/25
	6119	RPA3	replication protein A3, 14kDa	7p22	15/25
	5422	POLA1	polymerase (DNA directed), alpha 1, catalytic subunit	Xp22.1-p21.3	13/25
	5426	POLE	polymerase (DNA directed), epsilon, catalytic subunit	12q24.3	13/25
Multivariate	1278	COL1A2	collagen, type I, alpha 2	7q22.1	2/2
	1281	COL3A1	collagen, type III, alpha 1	2q31	2/2
	1285	COL4A3	collagen, type IV, alpha 3 (Goodpasture antigen)	2q36-q37	2/2
	1289	COL5A1	collagen, type V, alpha 1	9q34.2-q34.3	2/2
	1290	COL5A2	collagen, type V, alpha 2	2q14-q32	2/2
	1293	COL6A3	collagen, type VI, alpha 3	2q37	2/2
	2200	FBN1	fibrillin 1	15q21.1	2/2
	3910	LAMA4	laminin, alpha 4	6q21	2/2
	3915	LAMC1	laminin, gamma 1 (formerly LAMB2)	1q31	2/2
	80781	COL18A1	collagen, type XVIII, alpha 1	21q22.3	2/2
Basal	2197	FAU	Finkel-Biskis-Reilly murine sarcoma virus (FBR-MuSV) ubiquitously expressed	11q13	13/13
	6188	RPS3	ribosomal protein S3	11q13.3-q13.5	13/13
	6191	RPS4X	ribosomal protein S4, X-linked	Xq13.1	13/13
	6194	RPS6	ribosomal protein S6	9p21	13/13
	6203	RPS9	ribosomal protein S9	19q13.4	13/13
	6204	RPS10	ribosomal protein S10	6p21.31	13/13
	6206	RPS12	ribosomal protein S12	6q23.2	13/13
	6209	RPS15	ribosomal protein S15	19p13.3	13/13
	6222	RPS18	ribosomal protein S18	6p21.3	13/13
	6228	RPS23	ribosomal protein S23	5q14.2	13/13
	6229	RPS24	ribosomal protein S24	10q22	13/13
	6232	RPS27	ribosomal protein S27	1q21	13/13
	6235	RPS29	ribosomal protein S29	14q	13/13
Luminal B	990	CDC6	cell division cycle 6	17q21.3	4/4
	1017	CDK2	cyclin-dependent kinase 2	12q13	4/4
	1026	CDKN1A	cyclin-dependent kinase inhibitor 1A (p21, Cip1)	6p21.2	4/4
	4171	MCM2	minichromosome maintenance complex component 2	3q21	4/4
	4172	MCM3	minichromosome maintenance complex component 3	6p12	4/4
	4175	MCM6	minichromosome maintenance complex component 6	2q21	4/4
	4998	ORC1	origin recognition complex, subunit 1	1p32	4/4
	5000	ORC4	origin recognition complex, subunit 4	2q22-q23	4/4
	5426	POLE	polymerase (DNA directed), epsilon, catalytic subunit	12q24.3	4/4
	5427	POLE2	polymerase (DNA directed), epsilon 2, accessory subunit	14q21-q22	4/4
	5683	PSMA2	proteasome (prosome, macropain) subunit, alpha type, 2	7p13	4/4
	5688	PSMA7	proteasome (prosome, macropain) subunit, alpha type, 7	20q13.33	4/4
	5691	PSMB3	proteasome (prosome, macropain) subunit, beta type, 3	17q12	4/4
	5692	PSMB4	proteasome (prosome, macropain) subunit, beta type, 4	1q21	4/4
	5693	PSMB5	proteasome (prosome, macropain) subunit, beta type, 5	14q11.2	4/4
	5694	PSMB6	proteasome (prosome, macropain) subunit, beta type, 6	17p13	4/4
	5700	PSMC1	proteasome (prosome, macropain) 26S subunit, ATPase, 1	14q32.11	4/4
	5704	PSMC4	proteasome (prosome, macropain) 26S subunit, ATPase, 4	19q13.11-q13.13	4/4
	5705	PSMC5	proteasome (prosome, macropain) 26S subunit, ATPase, 5	17q23.3	4/4
	5706	PSMC6	proteasome (prosome, macropain) 26S subunit, ATPase, 6	14q22.1	4/4
	5708	PSMD2	proteasome (prosome, macropain) 26S subunit, non-ATPase, 2	3q27.1	4/4
	5709	PSMD3	proteasome (prosome, macropain) 26S subunit, non-ATPase, 3	17q21.1	4/4
	5710	PSMD4	proteasome (prosome, macropain) 26S subunit, non-ATPase, 4	1q21.3	4/4
	5713	PSMD7	proteasome (prosome, macropain) 26S subunit, non-ATPase, 7	16q22.3	4/4
	5717	PSMD11	proteasome (prosome, macropain) 26S subunit, non-ATPase, 11	17q11.2	4/4
	5718	PSMD12	proteasome (prosome, macropain) 26S subunit, non-ATPase, 12	17q24.2	4/4
	6119	RPA3	replication protein A3, 14kDa	7p22	4/4
	6233	RPS27A	ribosomal protein S27a	2p16	4/4
	8318	CDC45	cell division cycle 45	22q11.21	4/4
	8900	CCNA1	cyclin A1	13q12.3-q13	4/4
	10213	PSMD14	proteasome (prosome, macropain) 26S subunit, non-ATPase, 14	2q24.2	4/4
	23594	ORC6	origin recognition complex, subunit 6	16q12	4/4

#### Multivariate analysis on the entire combined dataset

Genes encoding for various types of collagens, fibrillin 1 and laminins were commonly distributed among the leading edges of the significant gene sets associated with bad prognosis (Table 9).

#### Univariate analysis on HER2 tumors

Since only one gene set was found significant in our analysis, a leading edge analysis could not be done on HER2 tumors.

#### Univariate analysis on the Basal tumors

Genes encoding for various ribosomal proteins were commonly distributed among the leading edges of the significant gene sets associated with bad prognosis (Table 9).

#### Univariate analysis on the Luminal A tumors

Genes encoding for various ribosomal proteins were commonly distributed among the leading edges of the significant gene sets associated with good prognosis (Table 8). Only one gene set (Reactome_Kinesins) was found significantly associated with bad prognosis in Luminal A tumors; hence a leading edge analysis could not be done for bad prognosis gene sets in Luminal A tumors.

#### Univariate analysis on Luminal B tumors

Genes encoding for various ribosomal proteins were commonly distributed among the leading edges of the gene sets significantly associated with good prognosis, while genes encoding for proteins involved in cell division (including cyclins, cyclin dependent kinases, proteasomes and replication factors) were commonly distributed among the leading edges of the significant gene sets associated with bad prognosis (Tables 8 and 9).

### IV) Results of the GSEA performed for the relationship between the AURKA module score and the other gene sets

In each data series, as well as in the combined dataset, a strong negative association was found between the AURKA module score and the gene sets related to the extracellular matrix (Table 10). The association of the gene sets representing translation did not reach statistical significance (FWER ≤ 0.05), though they were consistently expressed in a direction opposite to that of the AURKA score.

**Table 10:** GSEA results for association with the AURKA module score of the non-proliferation related gene sets found to be statistically significant on the univariate and multivariate analyses shown in Tables 3, 4 and 5.

	GSE 2034			GSE 5327			GSE 11121			GSE7390			Analysis on combined dataset	
Name of gene set	ES	NES	p-val	ES	NES	p-val	ES	NES	p-val	ES	NES	p-val	p-val(Stouffer)	FWER
KEGG_RIBOSOME	-0.665	-1.703	0.035	-0.485	-1.293	0.255	-0.556	-1.407	0.189	-0.602	-1.502	0.145	0.01	0.12
REACTOME_3_UTR_MEDIATED_TRANSLATIONAL_REGULATION	-0.619	-1.679	0.037	-0.435	-1.201	0.314	-0.503	-1.322	0.236	-0.543	-1.428	0.170	0.02	0.20
REACTOME_ACTIVATION_OF_THE_MRNA_UPON_BINDING_OF_THE_CAP_BINDING_COMPLEX_AND_EIFS_AND_SUBSEQUENT_BINDING_TO_43S	-0.498	-1.377	0.158	-0.333	-0.923	0.531	-0.401	-1.061	0.420	-0.391	-1.042	0.422	0.25	0.25
REACTOME_FORMATION_OF_THE_TERNARY_COMPLEX_AND_SUBSEQUENTLY_THE_43S_COMPLEX	-0.606	-1.524	0.086	-0.392	-1.005	0.478	-0.505	-1.238	0.299	-0.494	-1.197	0.328	0.12	0.35
REACTOME_INFLUENZA_VIRAL_RNA_TRANSCRIPTION_AND_REPLICATION	-0.547	-1.544	0.098	-0.341	-0.997	0.473	-0.433	-1.199	0.324	-0.456	-1.256	0.279	0.11	0.46
REACTOME_NONSENSE_MEDIATED_DECAY_ENHANCED_BY_THE_EXON_JUNCTION_COMPLEX	-0.549	-1.569	0.071	-0.361	-1.050	0.430	-0.472	-1.308	0.237	-0.519	-1.482	0.126	0.04	0.28
REACTOME_PEPTIDE_CHAIN_ELONGATION	-0.692	-1.766	0.019	-0.515	-1.348	0.214	-0.600	-1.501	0.131	-0.640	-1.590	0.078	0.003	0.03
REACTOME_SRP_DEPENDENT_COTRANSLATIONAL_PROTEIN_TARGETING_TO_MEMBRANE	-0.563	-1.652	0.065	-0.370	-1.116	0.368	-0.428	-1.228	0.301	-0.450	-1.281	0.252	0.06	0.39
REACTOME_TRANSLATION	-0.497	-1.577	0.085	-0.306	-0.991	0.461	-0.365	-1.104	0.378	-0.379	-1.156	0.321	0.13	0.26
STRUCTURAL_CONSTITUENT_OF_RIBOSOME	-0.577	-1.567	0.085	-0.388	-1.100	0.390	-0.461	-1.232	0.289	-0.487	-1.276	0.261	0.08	0.39
PID_INTEGRIN1_PATHWAY	-0.651	-1.864	0.014	-0.625	-1.862	0.022	-0.596	-1.714	0.046	-0.601	-1.693	0.050	<0.001	<0.001
PROTEINACEOUS_EXTRACELLULAR_MATRIX	-0.684	-2.157	0.001	-0.645	-2.094	0.001	-0.669	-2.062	0.001	-0.722	-2.241	0.001	<0.001	<0.001

Significant values (FWER ≤0.05) are shown in bold.(ES = Enrichment Score, NES = Normalized Enrichment Score, p-val = unadjusted p-value, p-val (Stouffer) = combined p-value using Stouffer's method, FWER = Family Wise Error Rate-adjusted p-value).

## Discussion

Most studies done on microarray data in breast cancer have found Cell cycle-related genes to have prognostic importance in breast cancer[9–14,58]; some studies have found the importance of immune related networks[9,16,19] and RNA splicing[9]. However, the prognostic importance of many processes may have been missed because: i) the aim of many of the studies was to find a gene signature predictive of outcome, and not the important cellular pathways and processes[15–22], and, ii) other studies only studied a few processes[12,59]; or did not include gene sets from many databases[23].

In contrast to the above studies, the present study focused on biological pathways and processes. Different datasets were combined to make the results more robust, with adjustment for study-specific differences being made by random effects models. Furthermore, batch effects were also treated as random effects, in order to adjust for any potential confounding effects thereof. The aim of the present study was to find processes and pathways associated with distant metastasis in the different molecular subtypes of breast cancer, as well as those processes associated with DMFS which were important in breast cancer taken as a whole.

The important pathways and processes were estimated from the entire list of genes ranked in order of their prognostic strength in each molecular subtype as well as overall breast cancer cases analyzed. In order not to miss out any important processes from the analysis, multiple gene sets were tested. These gene sets represent biological processes curated by multiple independent reliable sources, as mentioned in Methods above.

The appropriate design in the present case is a time-to-event analysis. Of the many gene set analysis implementations, we had to select one which could make sense of a time-to-event analysis and yet adjust for batch effects. Therefore it was felt suitable to perform mixed-effects Cox regression on each gene after adjustment for Centre and batch effects as a random variable, and then analyze the ranked list of the Cox regression coefficients.

A pre-ranked Gene Set Enrichment analysis (GSEA)[39,40] was preferred to analyze the ranked list, since the alternative(over-representation analysis[60] using the hypergeometric test) is limited by an arbitrary cut-off [61]value for gene selection and the statistical significance is also related to the size of the gene-set in the analysis. It may be noted that the standard GSEA currently lacks the software implementation for adjusting for batch effects, as well as being incapable of performing time-to event analysis.

To counteract the false positives, a strict criterion for significance was adopted by keeping the Family-wise Error Rate (FWER) ≤0.05. Pre-ranked GSEA results use gene set permutation rather than the more stringent phenotype permutation used by standard GSEA. The GSEA User Guide[62] suggests a cut-off of False Detection Rate (FDR) of 0.05 for analyses done with gene set permutation. This study consists of multiple independent analyses, which necessitate a stringent cut off to exclude false positives. An FDR of 0.05 means that in any given single analysis, the total number of false positives would be 5%; however, an FWER ≤0.05 means that in any given single analysis, the probability of having even one single false positive is not more than 5%. Therefore, we have used this more stringent threshold than suggested by the GSEA user guide. An even stricter cut off may have resulted in missing out many significant gene sets. To make the results of the pre-ranked GSEA robust, ten thousand permutations were used for finding the prognostically important gene sets. However, for the standard GSEA used to find gene sets associated with the AURKA module score, we were more interested in the general direction of association in each of the four data series, hence the standard recommended one thousand permutations were thought to confer sufficient robustness to the results.

Probably the most striking aspect of the results is the association of genes involved in protein translation with distant metastasis in breast cancer. Although the importance of protein translation in the molecular biology of cancers is well known, the association of this process with the prognosis of breast cancers is under appreciated. To our knowledge, this is possibly the first study to report on the prognostic importance of gene sets related to protein translation in breast cancer subtypes. On univariate analysis of the entire dataset, gene sets representing protein translation and the immune system were the only gene sets positively associated with survival (negatively associated with metastasis, i.e. having a good prognosis). As expected, gene sets representing cell cycle (or proliferation) were found to be negatively associated with survival in this analysis The leading edges of the significant translation-related gene sets in the different analyses were predominantly populated by genes encoding for ribosomal proteins.

On adjustment for proliferation in the multivariate analysis, protein translation ceased to retain its significance. On first impression, it may seem that the association of protein translation with DMFS is spurious. However, on univariate analysis of the individual molecular sub-types, it was found that these gene sets were associated with DMFS in the Luminal A and Basal sub-types, even though in such analyses proliferation-related gene sets were not found to be associated with DMFS (Tables 6 and 7). Thus, protein translation seems to have an association independent of cellular proliferation with survival in certain molecular sub-types of breast cancer.

It was interesting to note that the direction of association of gene sets related to protein translation with metastasis was different in the different sub-types. In the Luminal A and B tumors, protein translation was found to be negatively associated with metastasis (good prognosis), but in the Basal subtype, it was found to be positively associated with the same (poor prognosis). This was corroborated by the high degree of overlap of the leading edge genes which were associated with favorable prognosis in Luminal A and B tumors, while being associated with a poor prognosis in Basal tumors.

Why translation should have differing prognostic effects in different sub-types is unclear. We offer a provisional hypothesis that the prognostic effect of translation might be related to the deranged proteins in the cells. Increased protein translation in Basal tumors might imply greater translation of mutated proteins which cause further dysregulation of growth (leading to a worse prognosis); while increased protein translation in the Luminal tumors might imply an increased expression of normal proteins which tends the cells towards homeostasis (leading to a better prognosis). Thus, different protein translation derangement mechanisms may confer different prognostic significance in the various breast cancer subtypes.

To our knowledge, ours is the first study to link protein translation as a process/pathway to clinical outcomes in breast cancer. However, since ours is an exploratory analysis, our findings need to be confirmed on larger datasets. However, such a novel finding is intriguing and may well give us insights about the oncobiology and natural history of breast cancer and its sub-types.

The different molecular subtypes have different expressions of proliferation-related genes, as a result of which the range of expression of these genes are reduced in the different subtypes. Therefore, it is not surprising that, in our study, cell cycle has a relatively lower prognostic importance in many of the individual molecular subtypes, as compared to analysis on the entire breast cancer cases. Even then, proliferation retained its prime position in Luminal B tumors. A gene set representing Kinesins also attained significance in Luminal A tumors. The genes in the leading edge of this gene set are all kinesins which play an important role in mitosis.

Only a few studies have found the extracellular matrix (ECM) to be of prognostic importance in breast cancer[9,63,64]. One study[9] found that a module made of ECM genes was related to a prognostic gene signature. However, the above mentioned study finally selected Proliferation, Immune Response and RNA splicing as the main cellular events predictive of outcome in breast cancer. Some proteins related to ECM were identified as having prognostic importance in breast cancer by network analysis[63]; even then, the importance of the ECM was understated. Only one study [64] found gene sets related to ECM to be of primary importance.

In our study, the ECM-related gene sets did not show prognostic association with breast cancer on any univariate analysis. However, when adjusted for proliferation (in the multivariate analysis), there was a positive association between the ECM-related gene sets and metastasis, underscoring a negative prognostic significance of the expression of ECM-related genes in breast cancer. This was bolstered by the finding of collagen-related genes being over-represented in the 'leading edge' analysis performed separately.

We investigated as to why the prognostic effect of ECM-related genes remained hidden in the univariate analysis, while being apparent in the multivariate setting. Considering that the ECM-related gene sets showed association with metastasis after adjustment for proliferation (Table 5), we hypothesized that there could be suppression of the effect of ECM-related genes due to association of their expression with proliferation-related genes. To test this hypothesis, standard GSEA was performed to assess the relationship between the proliferation-related AURKA module score and all the gene sets in individual data series. This analysis confirmed a strong negative association between the AURKA score and the ECM gene sets, i.e. increased expression of ECM-related genes is associated with decreased expression of the proliferation-related genes, and vice versa (Table 10). Since proliferation is known to be one of the strongest markers of poor prognosis, hence the decreased expression of proliferation-related genes might mask the poor prognostic association of the increased expression of ECM-related genes, thus explaining our seemingly paradoxical results. This negative correlation between the expression of ECM-related genes and proliferation may also be a possible explanation for the prognostic non-significance of ECM-related genes in other studies which were not adjusted for proliferation. The overexpression of ECM and integrins being associated with poor prognosis (Table 5) makes biological sense, as they represent a part of the molecular mechanism of metastasis as is presently known[65].

A few caveats while interpreting the results have to be made: i) The study consisted of systemically untreated node negative females pooled from various studies which differed in their time periods from the 1980s through the 2000s. This has the advantage of having a more homogeneous cohort compared to random datasets having a variety of systemic treatments which would have complicated the analysis greatly. However, this criterion may itself lead to a dataset consisting of a biased population.

After combining data from the four series, 499 patients had ER positive tumors, while 295 had PR positive tumors–the data series did not allow for an interpretation whether these were mutually exclusive or overlapping in some way. However, despite being hormone receptor positive, none received any adjuvant hormonal therapy, the reasons for the exclusion from systemic treatment being unknown. Similarly, there were 325 patients who had T2 or larger tumors, but who, for unknown reasons, did not receive chemotherapy. We do not know whether the characteristic of the patients which led to their exclusion from systemic therapy would be a confounding variable in itself, as well as the possibility that since they did not receive any systemic therapy that itself could have affected their metastasis free survival in any way. It is certainly probable that the characteristics of patients and tumors (for instance, preferential evolutionary clonal selection[66]) in the different datasets changed with time as systemic treatment became more prevalent and few patients were recommended no systemic treatment.

ii) Since the population consisted of patients who were systemically untreated, the present study gives a good picture of the natural history of breast cancer unmodified by any systemic iatrogenic factor. However, the very same characteristic causes it to be unsuitable for the formation of a prognostic gene signature; prognosis being a much more complex end process, having highly multi-factorial stochastic and causal elements to it. At present, breast cancer patients are given a variety of treatments, and the responsiveness of these treatments may themselves be associated with gene expression[27–30].

iii) A third limitation of the study concerns the relatively small sample size of this study. Despite combining four large studies in the database, our study had 742 patients, and relatively little number of events of interest (i.e. distant metastases) had occurred at the time of data censoring. At the time of data censoring, a total of 200 events of interest had occurred– 52, 34, 47 and 59 for the Basal, HER2, Luminal A and Luminal B tumors respectively–which we feel might represent a complex interplay between differences in tumor biology and changing patterns of survival over the last few decades.

### Conclusion

This study, in addition to confirming the known prognostic role of proliferation in breast cancer, to the best of our knowledge, reports for the first time, the hitherto unappreciated prognostic effect of translation in breast cancer and its various molecular sub-types. It also shows the opposing prognostic association of the translation associated genes in the various breast cancer subtypes. Finally, the study highlights the independent prognostic significance of the genes related to integrin1 pathway and the extracellular matrix. This is possibly the first study to demonstrate the confounding effect of proliferation. Importantly, this study showed how the significant prognostic effects of biologically meaningful pathways and processes may be hidden by their association with a prognostically strong confounder (proliferation).

This study was undertaken as we felt that previous studies had methodological flaws, and no one has yet reported any results by combining different series in a single large dataset using a meta-analytic approach for the different breast cancer subtypes. The primary aim of this study was to better understand the processes and pathways affecting prognosis in breast cancer, and possibly identify novel pathways of interest for further analysis and hypothesis generation, rather than making a predictive signature. Confirmatory studies, preferably on large datasets, are needed to validate the findings of this study.

## Supporting Information

S1 Fig Boxplot of the entire microarray data before and after fRMA processing. The y-axis of the data before pre-processing is plotted after log 2 transformation. Data after fRMA preprocessing are log 2 transformed during pre-processing, hence no further log transformation is done.The different colors represent different data series (orange for GSE2034, blue for GSE5327, yellow for GSE11121 and green for GSE7390).
(TIFF)

S1 Folder A zipped folder containing the R script for the analysis along with the follow up data, instructions for analysis, and the.rnk files as well as text files having the GSEA parameters with which to reproduce the results of the present study. (ZIP)

S1 Spreadsheet Excel spreadsheet giving the full Leading edge results. 1 represents membership of the gene in the geneset, 0 denotes non-membership. A total denoting total number of leading edges the gene is part of is also given.
(XLSX)

S1 Table Supplementary Appendix 1 shows the AIC values for each Cox Regression model used in each analysis. In each analysis, Gene Expression was the fixed effects variable. In analysis (b) the AURKA module score was an additional fixed effects variable. For any given analysis, the particular model having the least median AIC values (indicated in bold red) was chosen as the best model, for the respective analysis, to be used in the pre-ranked GSEA. (RE = Random effects).
(DOCX)

S2 Table Summary of results obtained in the six analyses showing the association of various gene sets with distant metastasis. (DOCX)
