# Supplementary figures and images for: Association of Protein Translation and Extracellular Matrix Gene Sets with Breast Cancer Metastasis: Findings Uncovered on Analysis of Multiple Publicly Available Datasets Using Individual Patient Data Approach
Source: PLoS One. 2015 Jun 16;10(6):e0129610. doi: 10.1371/journal.pone.0129610 (PMC4469303; doi:10.1371/journal.pone.0129610)

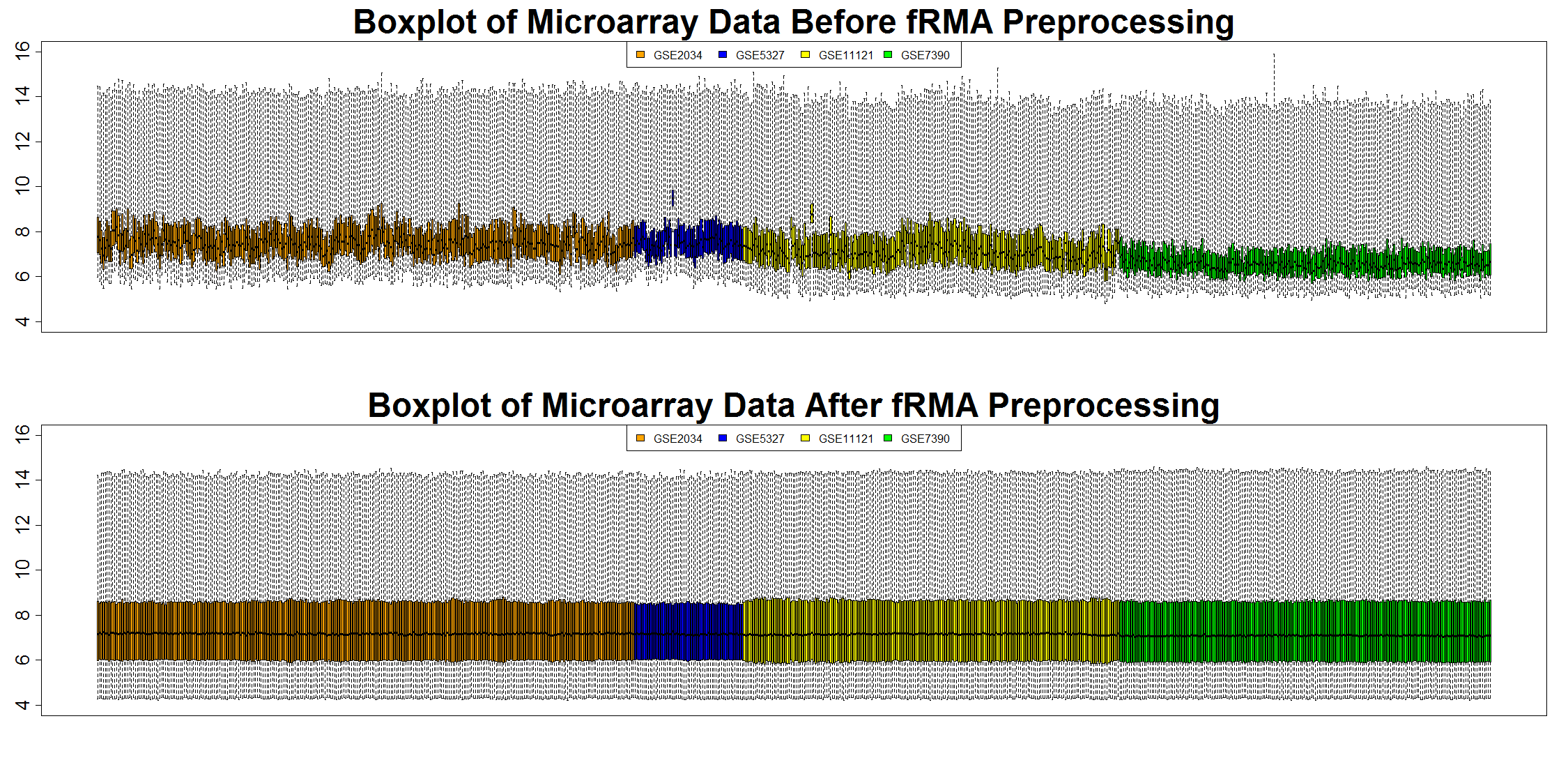

Supplement: S1 Fig — The y-axis of the data before pre-processing is plotted after log 2 transformation. Data after fRMA preprocessing are log 2 transformed during pre-processing, hence no further log transformation is done.The different colors represent different data series (orange for GSE2034, blue for GSE5327, yellow for GSE11121 and green for GSE7390). (TIFF) [file pone.0129610.s001.tiff]
