# Supplementary material for: Association of Protein Translation and Extracellular Matrix Gene Sets with Breast Cancer Metastasis: Findings Uncovered on Analysis of Multiple Publicly Available Datasets Using Individual Patient Data Approach
Source: PLoS One. 2015 Jun 16;10(6):e0129610. doi: 10.1371/journal.pone.0129610 (PMC4469303; doi:10.1371/journal.pone.0129610)
Supplement: S1 Table — In each analysis, Gene Expression was the fixed effects variable. In analysis (b) the AURKA module score was an additional fixed effects variable. For any given analysis, the particular model having the least median AIC values (indicated in bold red) was chosen as the best model, for the respective analysis, to be used in the pre-ranked GSEA. (RE = Random effects). (DOCX) [file pone.0129610.s004.docx]

**Supplementary Table 1:**

|  |  | **Model (i): Batch as RE Variable** | **Model (ii): Data Series as RE Variable** | **Model (iii): Centre as RE Variable** | **Model (iv): Batch nested in Data Series as RE Variable** | **Model (v): Batch nested in Centre as RE Variable** | **Model (vi): No RE variable** |
| --- | --- | --- | --- | --- | --- | --- | --- |
| **Analysis** | **AIC value obtained for each model** | | | | | | |
| **(a) Univariate analysis, on combined dataset** | | | | | | | |
|  | Minimum | 2434 | 2496 | 2502 | 2433 | 2434 | 2509 |
|  | 1^st^ quartile | 2475 | 2544 | 2549 | 2475 | 2475 | 2558 |
|  | **Median** | **2477** | 2547 | 2551 | 2477 | 2478 | 2562 |
|  | Mean | 2475 | 2545 | 2550 | 2476 | 2476 | 2559 |
|  | 3^rd^ quartile | 2478 | 2548 | 2552 | 2478 | 2478 | 2563 |
|  | Maximum | 2479 | 2548 | 2553 | 2478 | 2480 | 2564 |
| **(b) Multivariate analysis, on combined dataset** | | | | | | | |
|  | Minimum | 2415 | 2479 | 2486 | 2417 | 2417 | 2484 |
|  | 1^st^ quartile | 2446 | 2513 | 2522 | 2447 | 2447 | 2528 |
|  | **Median** | **2448** | 2515 | 2524 | 2448 | 2448 | 2531 |
|  | Mean | 2447 | 2514 | 2523 | 2447 | 2448 | 2529 |
|  | 3^rd^ quartile | 2448 | 2516 | 2525 | 2448 | 2449 | 2532 |
|  | Maximum | 2448 | 2516 | 2526 | 2449 | 2449 | 2532 |
| **(c) Univariate analysis on HER2 subtype** | | | | | | | |
|  | Minimum | 278.0 | 278.6 | 278.6 | 280.0 | 280.0 | 276.6 |
|  | 1^st^ quartile | 294.9 | 298.8 | 299.2 | 296.6 | 296.7 | 297.3 |
|  | **Median** | **296.1** | 300.2 | 300.6 | 297.8 | 298.0 | 298.7 |
|  | Mean | 295.4 | 299.3 | 299.7 | 297.0 | 297.3 | 297.8 |
|  | 3^rd^ quartile | 296.6 | 300.7 | 301.2 | 298.3 | 298.6 | 299.2 |
|  | Maximum | 296.8 | 300.9 | 301.4 | 298.4 | 298.7 | 299.4 |
| **(d) Univariate analysis on Basal subtype** | | | | | | | |
|  | Minimum | 491.7 | 497.1 | 497.1 | 493.7 | 493.6 | 495.1 |
|  | 1^st^ quartile | 504.6 | 510.5 | 510.7 | 506.5 | 506.5 | 508.7 |
|  | **Median** | **505.6** | 511.6 | 511.8 | 507.5 | 507.5 | 509.8 |
|  | Mean | 505.0 | 510.9 | 511.1 | 506.9 | 506.9 | 509.1 |
|  | 3^rd^ quartile | 506.0 | 512.1 | 512.2 | 508 | 508 | 510.2 |
|  | Maximum | 506.1 | 512.2 | 512.3 | 508.1 | 508.1 | 510.3 |
| **(e) Univariate analysis on Luminal A subtype** | | | | | | | |
|  | Minimum | 469.6 | 476.6 | 475.9 | 467.8 | 467.4 | 484.1 |
|  | 1^st^ quartile | 493.1 | 505.2 | 504.7 | 490.1 | 489.7 | 510.4 |
|  | **Median** | 494.8 | 506.4 | 505.8 | 491.1 | 490.7 | 513.2 |
|  | Mean | 493.9 | 505.6 | 505.1 | 490.4 | 490 | 511.8 |
|  | 3^rd^ quartile | 495.6 | 506.8 | 506.3 | 491.5 | 491.1 | 514.5 |
|  | Maximum | 496 | 507 | 506.4 | 491.8 | 491.3 | 514.8 |
| **(f) Univariate analysis on Luminal B subtype** | | | | | | | |
|  | Minimum | 533.1 | 539.3 | 539.3 | 532.6 | 533 | 537.3 |
|  | 1^st^ quartile | 552 | 561.5 | 562.5 | 552.6 | 553.1 | 564.4 |
|  | **Median** | 553.1 | 563.1 | 564.1 | 553.9 | 554.3 | 566 |
|  | Mean | 552.3 | 562.2 | 563.2 | 553.1 | 553.6 | 565.1 |
|  | 3^rd^ quartile | 553.5 | 563.8 | 564.8 | 554.4 | 554.8 | 566.7 |
|  | Maximum | 553.7 | 564.1 | 565 | 554.6 | 555 | 566.9 |
