# Supplementary material for: Association of Protein Translation and Extracellular Matrix Gene Sets with Breast Cancer Metastasis: Findings Uncovered on Analysis of Multiple Publicly Available Datasets Using Individual Patient Data Approach
Source: PLoS One. 2015 Jun 16;10(6):e0129610. doi: 10.1371/journal.pone.0129610 (PMC4469303; doi:10.1371/journal.pone.0129610)
Supplement: S2 Table — (DOCX) [file pone.0129610.s005.docx]

**S2 Table: Summary of results obtained in the six analyses showing the association of various gene sets with distant metastasis.**

(NS=Not significant at FWER-adjusted p-value ≤0.05)

|  | **Analysis** | | | | | | |
| --- | --- | --- | --- | --- | --- | --- | --- |
| **Association of gene sets with metastasis**  **(Prognosis)** |  | **(a) Univariate, combined dataset** | **(b) Multivariate, combined dataset** | **(c) Univariate, HER2** | **(d) Univariate, Basal** | **(e) Univariate, Luminal A** | **(f) Univariate, Luminal B** |
|  | **Translation related gene sets** | **Negative**  **(good)** | NS | NS | **Positive**  **(poor)** | **Negative**  **(good)** | **Negative**  **(good)** |
|  | **Cell cycle/ Proliferation related gene sets** | **Positive**  **(poor)** | NS | NS | NS | **Positive**  **(poor)** | **Positive**  **(poor)** |
|  | **Immune system related gene sets** | NS | NS | **Negative**  **(good)** | NS | NS | NS |
|  | **ECM related gene sets** | NS | **Positive**  **(poor)** | NS | NS | NS | NS |
